# A systematic review and meta-analysis of effectiveness and safety of therapy for overactive bladder using botulinum toxin A at different dosages

**DOI:** 10.18632/oncotarget.20056

**Published:** 2017-08-07

**Authors:** Hui-Yun Gu, Ju-Kun Song, Wen-Jun Zhang, Jin Xie, Qi-Sheng Yao, Wen-Jing Zeng, Chao Zhang, Yu-Ming Niu

**Affiliations:** ^1^ Center for Evidence-Based Medicine and Clinical Research, Taihe Hospital, Hubei University of Medicine, Shiyan 442000, China; ^2^ Department of Oral and Maxillary Surgery, Guizhou Provincial People’s Hospital, Guiyang 550002, China; ^3^ Department of Ultrasound, Taihe Hospital, Hubei University of Medicine, Shiyan 442000, China; ^4^ Administrative Office, Taihe Hospital, Hubei University of Medicine, Shiyan 442000, China; ^5^ Department of Urinary Surgery, Taihe Hospital, Hubei University of Medicine, Shiyan 442000, China; ^6^ Department of Anesthesiology, Taihe Hospital, Hubei University of Medicine, Shiyan 442000, China

**Keywords:** overactive bladder, botulinum toxin A, urinary incontinence, incontinence quality of life, meta-analysis

## Abstract

**Purpose:**

To assess the effectiveness and safety of botulinum toxin A (BTX-A) at different dosages for overactive bladder (OAB).

**Materials and Methods:**

The MEDLINE, EMBASE, and Cochrane Controlled Trials Register databases were searched through November 3, 2016 to identify relevant randomized controlled trials (RCTs).

**Results:**

Eleven studies were identified in this meta-analysis. Compared with placebo, the urinary incontinence (UI) episodes per week as the primary outcomes, urodynamic parameters including maximum cystometric capacity (MCC), and maximum detrusor pressure (MDP) for neurogenic detrusor overactivity (NDO) at 6 weeks, and for idiopathic detrusor overactivity (IDO) at 36 weeks were evaluated. These and other outcomes for effectiveness of BTX-A at different dosages in two observation periods indicate that a dose greater than 50 U is significantly more effective for certain symptoms of OAB compared with placebo. However, there were no significant differences between some dosages. Compared with placebo, the outcomes of total adverse events for NDO and for IDO show that doses of 300 U and 200 U for NDO are associated with more complications.

**Conclusions:**

In consideration that the treatments of BTX-A were with minimal, local, and manageable adverse effects, this meta-analysis demonstrates that BTX-A 200 U is recommended for management of NDO for short-term treatment for there was no significant difference from the larger dose of 300U. The short-term efficacies of BTX-A for IDO remain to be investigated.

## INTRODUCTION

Overactive bladder (OAB) is a multifactorial and chronic disease with lower urinary tract symptoms, and is defined by the International Continence Society as urgency with or without urge urinary incontinence, usually accompanied by frequency and nocturia, but not caused by urinary tract infection (UTI) [1]. The prevalence of OAB is approximately 12–19% in both men and women [2], and has a detrimental effect on the quality of life and social functioning [3]. OAB includes detrusor instability and detrusor hyperreflexia, with a relatively high incidence of neurogenic detrusor overactivity (NDO) due to spinal cord injury (SCI) or other neurologic conditions such as multiple sclerosis (MS) [4]. Idiopathic detrusor overactivity (IDO) is another common type of OAB. OAB can lead to urinary incontinence and frequency and changes in urodynamic parameters including bladder capacity, compliance, and other important functions [5, 6]. Current first-line treatment for OAB is antimuscarinic medicine [7]. However, long-term antimuscarinic treatment is suboptimal, because of inadequate effectiveness and bothersome side effects [8, 9].

Botulinum toxin A (BTX-A) produced by Clostridium botulinum is a potential alternative. BTX-A is a neuromodulator that inhibits the release of acetylcholine and other neurotransmitters by binding to synaptic vesicle glycoprotein 2A and reduces muscle spasticity [10, 11]. The use of BTX-A for NDO was first reported by Schurch [12], and Smith [13] investigated its effect in an animal model. Based on its mechanism and subsequent studies, BTX-A became a second line medicine for NDO, as recommended by a European consensus report [14] and the Food and Drug Administration.

Several relevant meta-analyses [15–21] had been conducted for NDO. In these meta-analyses, some studies compared different dosages of BTX-A with placebo at only one time point after BTX-A injection [15, 20, 21], while others compared pooled dosages with placebo [16, 17]; however, there was a lack of comprehensive analysis and consideration of the influence of the dosages on effectiveness outcomes. One meta-analysis [19] compared the grouped data of the same outcome indicators at different time points, which could produce errors and interfere with correct conclusions, thus reducing the reliability and value of the study. For IDO, although, sacral neuromodulation is the approved second-line treatment [22] and the effectiveness of BTX-A for IDO has been demonstrated in a number of uncontrolled studies [23–29] and in small Randomized controlled trials (RCTs) [30, 31], the effectiveness and safety is still under evaluation. To resolve these questions about effectiveness and safety, this study will examine outcomes using different dosages of BTX-A for NDO and IDO, and will evaluate the evidence for optimal doses in the clinical management of NDO and IDO.

## RESULTS

### Study selection and characteristics of individual studies

**Figure 1:**
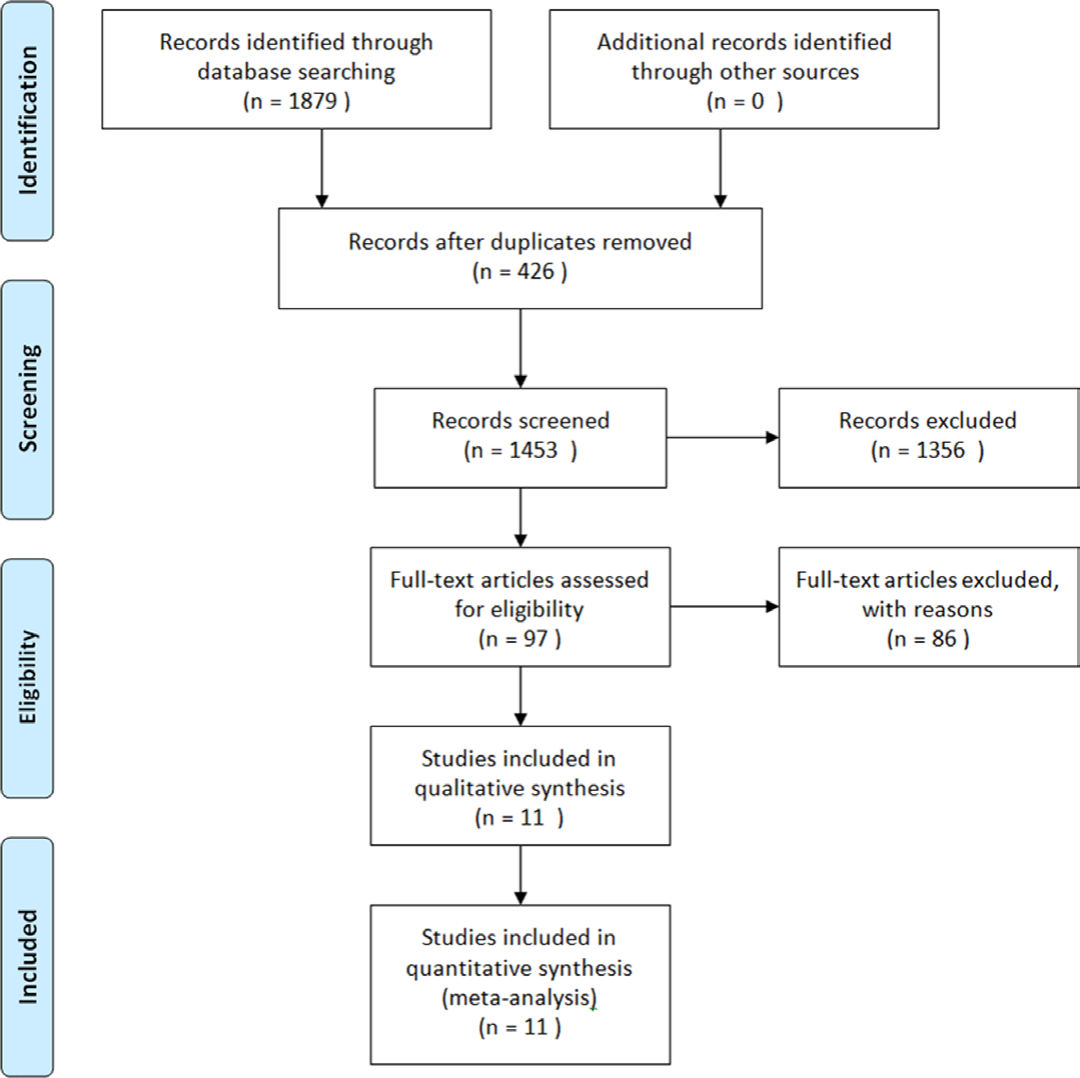
A flow diagram of the study selection process

A total of 1,418 studies were initially identified for use in our meta-analysis. Of these, 203 were excluded as duplicates. Of the remaining 1,215 potential articles, 1,131 were excluded by reading titles and abstracts, and the full texts of 84 articles were independently read by two authors. Eventually, 11 articles [22, 32–41] were included in our meta-analysis (Figure 1). The characteristics of the individual study are presented in Table 1.

**Table 1:** Characteristics of individual study

First(author)	Year	Region	No.of patiens (female)	Ages mean (SD)	Design	Classification of urinary incontinence	Basic diseases	Intervention	Follow-up (weeks)
Schurch [41]	2005	Switzerland	59 (23)	41	Randomized, doubled-blind	NDO	MS:6, SCI:53	Group 1: BTX-A 300U (19)Group 2: BTX-A 200U (19) Group 3: Placebo (21)	6
Dmochowski [34]	2010	USA	313 (288)	58.8	Randomized, doubled-blind	IOAB	NA	Group 1: BTX-A 50U (56) Group 2: BTX-A 100U (55) Group 3: BTX-A 150U (50) Group 4: BTX-A 200U (52) Group 5: BTX-A 300U(55) Group 6: Placebo (43)	NA
Cruz [33]	2011	Portugal	275 (155)	46(13.1), 44.4(13.9), 46.9(13.4)	Randomized, doubled-blind	NDO	MS:154, SCI:121	Group 1: BTX-A 200U(92) Group 2: BTX-A 300U(91) Group 3: Placebo (92)	2, 6, 12
Rovner [40]	2011	USA	313 (288)	58.8	Randomized, doubled-blind	IOAB	NA	Group 1: BTX-A 50U(57) Group 2: BTX-A 100U(54) Group 3: BTX-A 150U(49) Group 4: BTX-A 200U(53) Group 5: BTX-A 300U(56) Group 6: Placebo (44)	12, 36
Denys [22]	2012	France	199 (87)	62.3, 61.7	Randomized, doubled-blind	IOAB	NA	Group 1: BTX-A 50U(23) Group 2: BTX-A 100U(23) Group 3: BTX-A 150U(30) Group 4: Placebo (31)	12
Ginsberg [36]	2012	USA	416 (245)	46(13)	Randomized, doubled-blind	NDO	MS:227, SCI:189	Group 1: BTX-A200U(135) Group 2: BTX-A300U(127) Group 3: Placebo (145)	6
Ginsberg [35]	2013	USA	381 (311)	49.7(12.1), 49.9(10.7), 50.2(10.7)	Randomized, doubled-blind	NDO	MS:381, SCI:310	Group 1: BTX-A 200U(227) Group 2: BTX-A 300U(223) Group 3: Placebo (241)	6
Nitti [38]	2013	USA	557 (497)	61.7(12.7), 61(13.1)	Randomized, doubled-blind	IOAB	NA	Group 1: BTX-A100U (278) Group 2: Placebo (272)	12
Rovner [39]	2013	USA	691(400)	45.9, 45.6, 46.2	Randomized, doubled-blind	NDO	MS:103, SCI:138	Group 1: BTX-A 200U(227) Group 2: BTX-A 300U(223) Group 3: Placebo (241)	6
Kennelly [37]	2013	USA	387 (233)	46.4	Randomized, doubled-blind	NDO	SCI, MS	Group 1: BTX-A 300U(185) Group 2: BTX-A 200U(202)	6
Abdelwahab [32]	2015	Egypt	80 (63)	31.35, 30.22	Randomized, doubled-blind	IOAB	NA	Group 2: BoNTA 200U (40) Group 1: BTX-A 100U (40)	12, 36

NDO: Neurogenic detrusor overactivity, IDO: Idiopathic detrusor overactivity, MS: Multiple sclerosis, SCI: Spinal cord injury, BTX-A: Botulinum toxin A, NA: Not available.

### Quality of the individual studies

The results of the risk of bias assessment are shown in Table 2. Eleven RCTs included in this meta-analysis were characterized as high quality studies.

**Table 2:** Quality assessment of individual study

Author	Year	Random sequence generation	Allocation concealment	Blinding of participants and personnel	Blinding of outcome assessment	Incomplete outcome data	Selective reporting	Other
Schurch [41]	2005	low	low	low	low	low	low	unclear
Dmochowski [34]	2010	low	unclear	low	low	low	low	unclear
Cruz [33]	2011	low	low	low	low	low	low	unclear
Rovner [40]	2011	low	low	unclear	low	low	low	unclear
Denys [22]	2012	low	unclear	low	low	low	low	unclear
Ginsberg [36]	2012	low	low	low	low	low	low	unclear
Ginsberg [35]	2013	low	low	low	low	low	low	unclear
Nitti [38]	2013	low	low	unclear	low	low	low	unclear
Rovner [39]	2013	low	low	low	low	low	low	unclear
Kennelly [37]	2013	low	low	low	low	low	low	unclear
Abdelwahab [32]	2015	low	unclear	low	low	low	low	unclear

### Effectiveness

### Urinary incontinence (UI) episodes per week

### UI episodes per week of NDO based on short-term observation at 2 weeks

**Figure 2:**
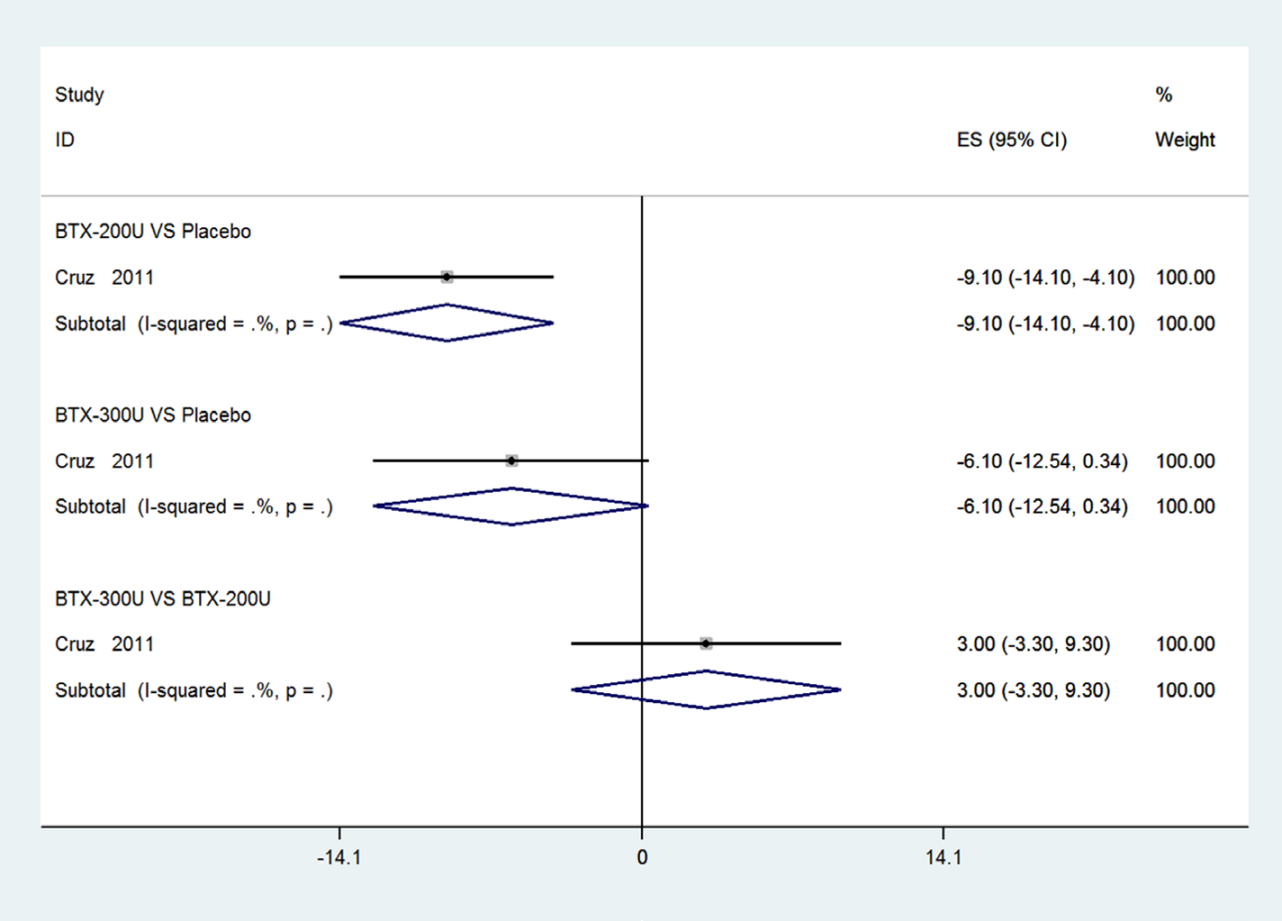
Forest plot of the changes of UI episodes per week of NDO at 2 weeks

One RCT [33] comprised BTX-A groups (200 U and 300 U) and a placebo group was identified and included to evaluate outcomes at 2 weeks. Compared with placebo in a fixed-effects model, Figure 2 show that BTX-A 200 U for NDO significantly reduced UI episodes per week (weighted mean difference (WMD): -9.10, 95% confidence interval (CI): −14.10 to -4.10, *I*^2^ = Not available (NA), *P* = NA), but the result for 300 U vs. placebo showed no significant reduction in UI episodes per week of NDO (WMD: −6.10, 95% CI: −12.54 to 0.34, *I*^2^ = NA, *P* = NA). However, there were no differences between BTX-A 300 U and 200 U in the fixed-effects model (WMD: 3.00, 95% CI: −3.30 to 9.30, *I*^2^ = NA, *P* = NA).

### UI episodes per week of NDO based on short-term observation at 6 weeks

**Figure 3:**
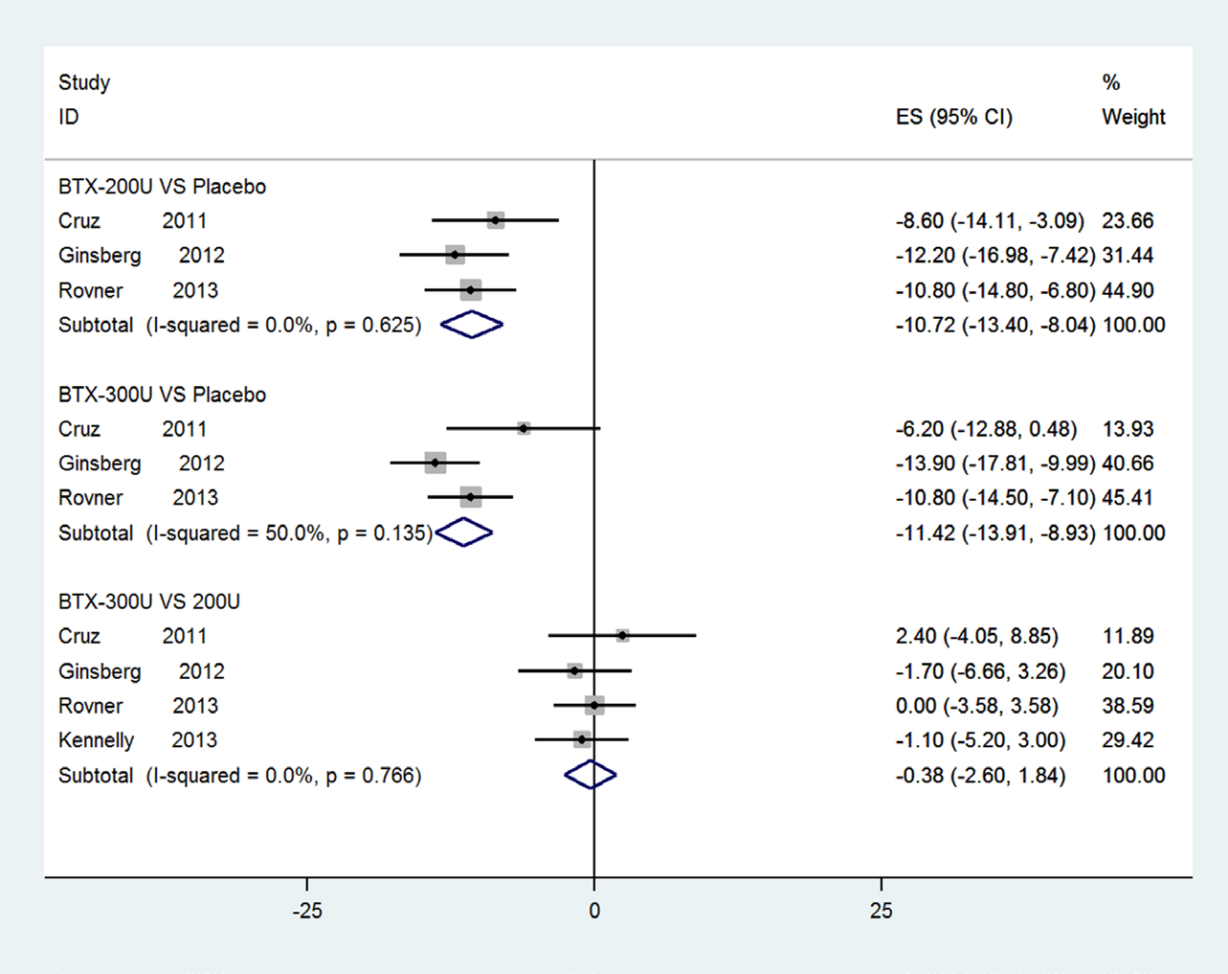
Forest plot of the changes of UI episodes per week of NDO at 6 weeks

Four studies [33, 36, 37, 39] comprised of BTX-A groups (200 U and 300 U) and a placebo group were identified and included to evaluate the outcomes at 6 weeks. Figure 3 show significant reductions in UI episodes per week of NDO (BTX-A 300 U vs. placebo, WMD: −11.42, 95% CI: −13.91 to −8.93, *I*^2^ = 50.0%, *P* = 0.135; BTX-A 200 U vs. placebo, WMD: −10.72, 95% CI: −13.40 to −8.04, *I*^2^ = 0.0%, *P* = 0.626). However, the differences between 300 U and 200 U at 6 weeks were not statistically significant in the fixed-effects model (WMD: −0.38, 95% CI: −2.60 to 1.84, *I*^2^ = 0.0%, *P* = 0.765).

### UI episodes per week of NDO based on short-term observation at 12 weeks

**Figure 4:**
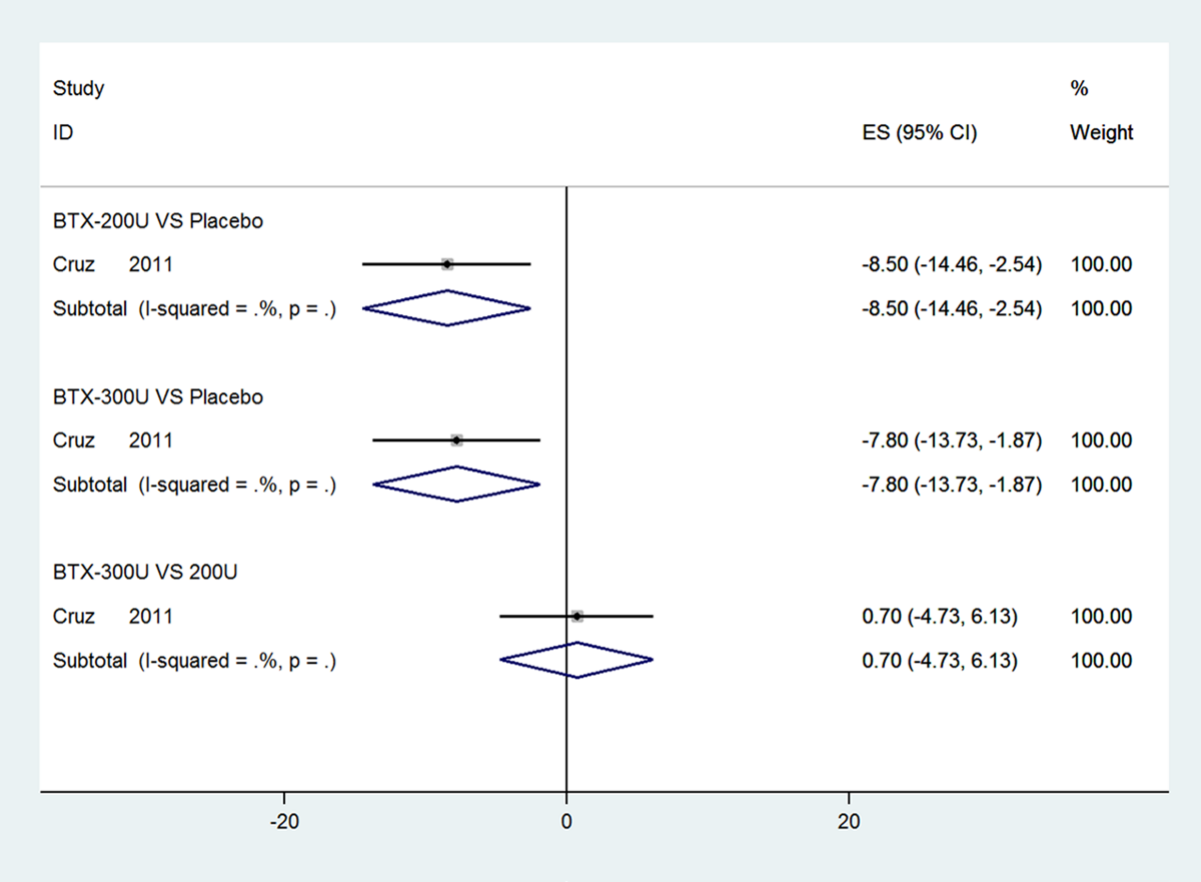
Forest plot of the changes of UI episodes per week of NDO at 12 weeks

One RCT [33] with data for UI episodes per week at 12 weeks was evaluated. Figure 4 show significant reductions in UI episodes per week of NDO in both 200 U and 300 U BTX-A groups compared with placebo at 12 weeks (WMD: −8.50, 95% CI: −14.46 to −2.54, *I*^2^ = NA, *P* = NA; WMD: −7.80, 95% CI: −13.73 to −1.87, *I*^2^ = NA, *P* = NA, respectively.). The outcome of subgroup analysis showed no significant differences between the BTX-A groups (WMD: 0.70, 95% CI: −4.73 to 6.13, *I*^2^ = NA, *P* = NA).

### Maximum cystometric capacity (MCC)

### MCC of NDO based on short-term observation at 6 weeks

**Figure 5:**
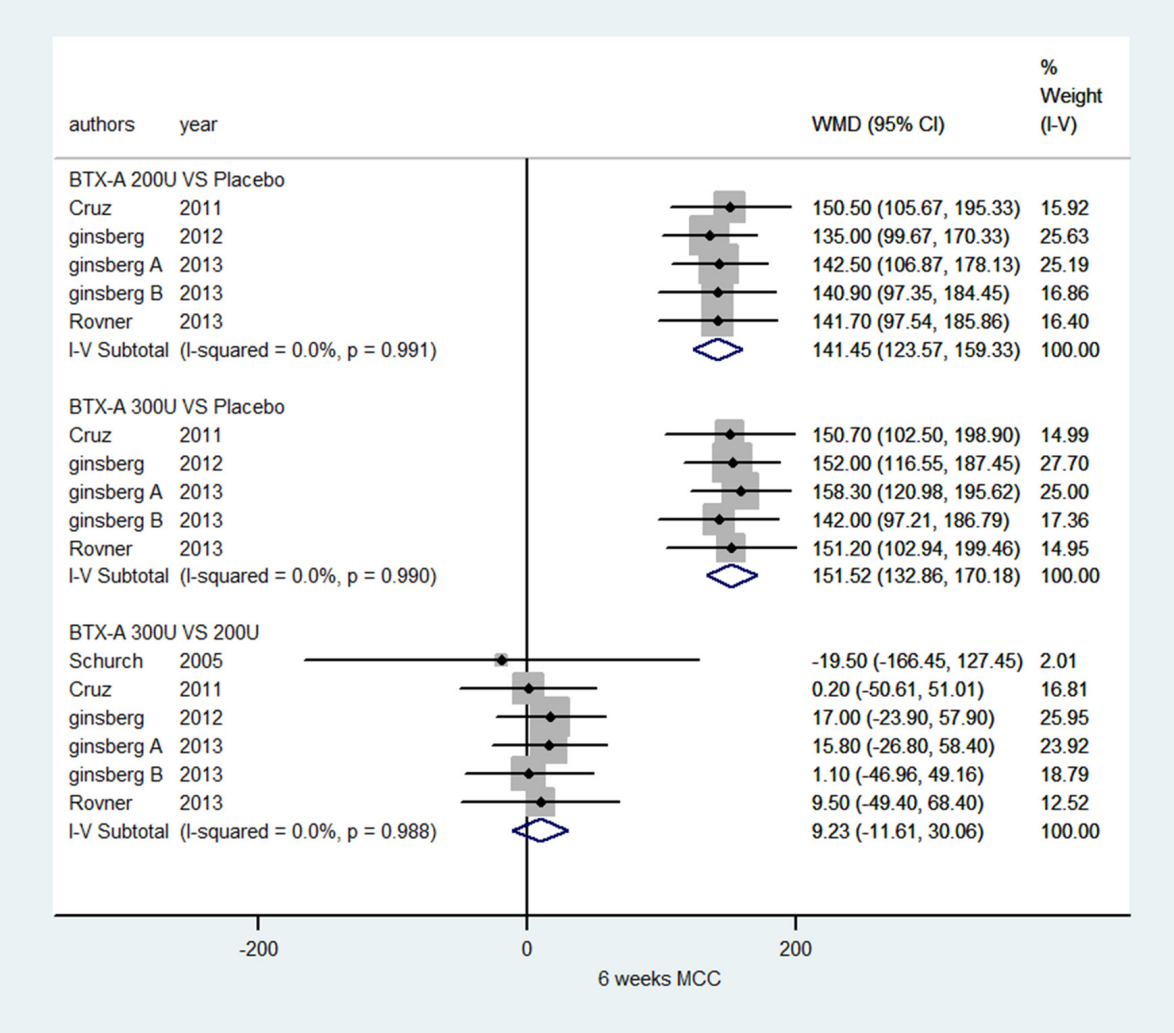
Forest plot of the changes of MCC of NDO at 6 weeks

Five RCTs [33, 35, 36, 39, 41] reporting data for MCC were collected in our study. Figure 5 show statistically significant improvement in MCC of NDO at 6 weeks in a comparison of BTX-A 300 U and 200 U with placebo (WMD: 151.52, 95% CI: 132.86 to170.18, I^2^ = 0.0%, *P* = 0.990; WMD: 141.45, 95% CI: 123.57 to 159.33, *I*^2^ = 0.0, *P* = 0.991, respectively). Significant improvements were not seen in a comparison of BTX-A 300 U vs. 200 U (WMD: 9.23, 95% CI: −11.61 to 30.06, *I*^2^ = 0.0%, *P* = 0.988).

### MCC of IDO based on short-term observation at 12 week

**Figure 6:**
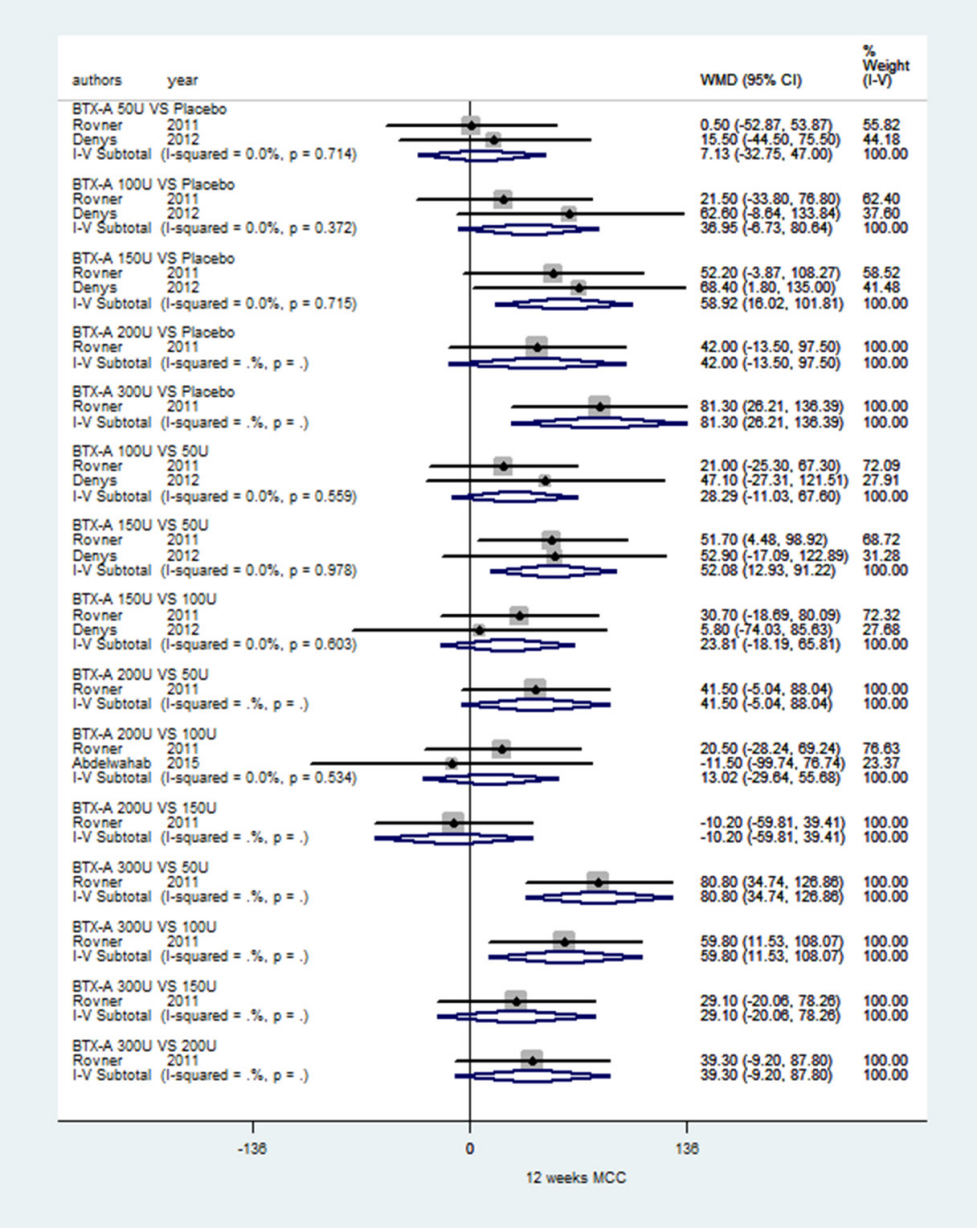
Forest plot of the changes of MCC of IDO at 12 weeks

Three RCTs [22, 32, 40] using 5 different doses of BTX-A vs. placebo performed an analysis of MCC; the subgroup details are shown in Figure 6. Figure 6 show that significant improvement was only seen in subgroups of BTX-A 150 U and 300 U vs. placebo, BTX-A 150 U vs. 50 U, BTX-A 300 U vs. 50 U, and BTX-A 300 U vs. 100 U for IDO (WMD: 58.92, 95% CI: 16.02 to 101.81,*I*^2^ = 0.0%, *P* = 0.715; WMD: 81.30, 95% CI: 26.21 to 136.39, *I*^2^ = NA, *P* = NA; WMD: 52.08, 95% CI:12.93 to 91.22, *I*^2^ = 0.0%, *P* = 0.978; WMD: 80.80, 95% CI:34.74 to 126.86, *I*^2^ = NA, *P* = NA; WMD: 59.80, 95% CI:11.53 to 108.07, *I*^2^ = NA, *P* = NA). However, other subgroup results showed no statistical significance.

### MCC of IDO based on long-term observation at 36 weeks

**Figure 7:**
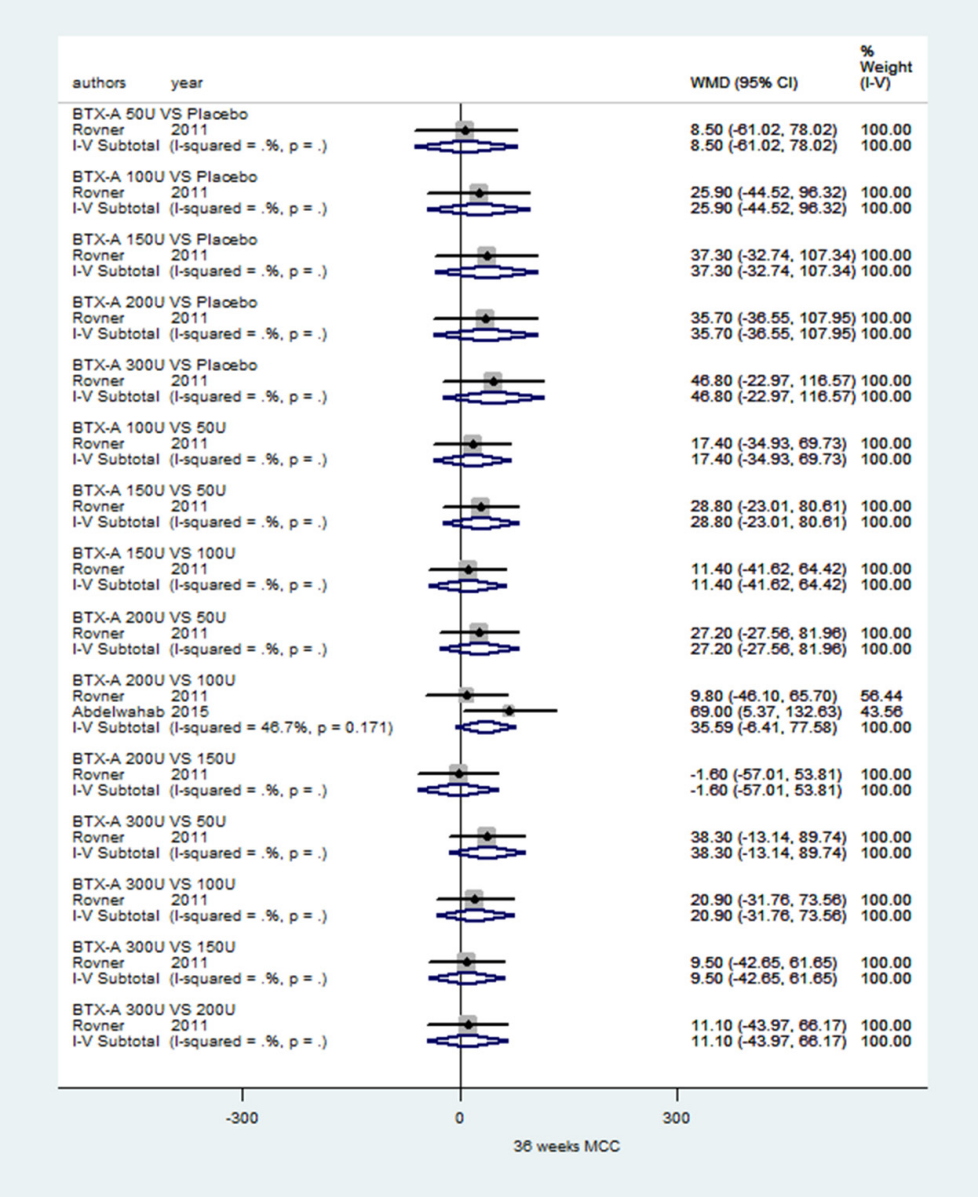
Forest plot of the changes of MCC of IDO at 36 weeks

Two RCTs [32, 40] met the criteria. As shown in Figure 7, no subgroup results showed statistical significance in a fixed-effects model at 36 weeks.

### The volume per void

### The volume per void of NDO based on short-term observation at 2 weeks

One RCT [33] was included for the volume per void. As shown in Supplementary Table 1, it is clear that both BTX-A 300 U and 200 U for NDO were superior to placebo at 2 weeks in the fixed-effects model (BTX-A 300 U vs. placebo, WMD: 49.90, 95% CI: 17.54 to 82.26, *I*^2^ = NA, *P* = NA; BTX-A 200 U vs. placebo, WMD: 42.60, 95% CI: 14.49 to 70.71, *I*^2^ = NA, *P* = NA). Nevertheless, significant differences were not observed at 2 weeks between 300 U and 200 U (WMD: 7.30, 95% CI: -30.28 to 44.88, *I*^2^ = NA, *P* = NA).

### The volume per void of NDO based on short-term observation at 6 weeks

Three RCTs [33, 37, 39] were included for the volume per void. As shown in Supplementary Table 1, it is clear that both BTX-A 300 U and 200 U for NDO were superior to placebo at 6 weeks in the fixed-effects model (BTX-A 300 U vs. placebo, WMD: 100.73, 95% CI: 83.24 to 118.22, *I*^2^ = 0.0%, *P* = 0.328; BTX-A 200 U vs. placebo, WMD: 92.68, 95% CI: 76.22 to 109.14, *I*^2^ = 0.0%, *P* = 0.982). Nevertheless, significant differences were not observed at 6 weeks between 300 U and 200 U (WMD: 4.72, 95% CI: −12.12 to 21.57, *I*^2^ = 0.0%, *P* = 0.663).

### The volume per void of NDO and IDO based on short-term observation at 12 weeks

Three RCTs [22, 33, 38] were included for analysis of the volume per void. In accordance with doses of BTX-A and the types of OAB, 9 subgroup analyses were conducted using a fixed-effects model (BTX-A 100 U vs. placebo, *I*^2^ = 42.0%, *P* = 0.189; others, I^2^ = NA, *P* = NA). Supplementary Tables 1 and 2 show significant improvement in the volume per void for BTX-A 100 U, 150 U for IDO, 200 U, 300 U for NDO compared with placebo, and for 100 U vs. 50 U for IDO (WMD: 34.48, 95% CI: 16.73 to 52.23, *I*^2^ = 42.0%, *P* = 0.189; WMD: 81.00, 95% CI: 7.98 to 154.02, *I*^2^ = NA, *P* = NA; WMD: 83.70, 95% CI: 54.54 to 112.86, *I*^2^ = NA, *P* = NA; WMD: 78.80, 95% CI: 46.23 to 111.37, *I*^2^ = NA, *P* = NA; WMD: 34.40, 95% CI: 3.79 to 65.01, *I*^2^ = NA, *P* = NA, respectively). The other subgroups showed no significant results.

### Maximum detrusor pressure (MDP)

### MDP of NDO based on short-term observation at 6 weeks

Five RCTs [33, 35, 36, 39, 41] included MDP data, generating 3 subgroups. As shown in Supplementary Table 1, compared with placebo in the fixed-effects model, BTX-A 300 U and 200 U for NDO both showed significant declines in MDP (WMD: −31.31, 95% CI: -35.79 to −26.84, *I*^2^ = 0.0%, *P* = 0.679; WMD: −33.01, 95% CI: −37.75 to −28.27, *I*^2^ = 0.0%, *P* = 0.998, respectively). However, there were no significant differences between BTX-A 300 U and 200 U for NDO (WMD: 1.16, 95% CI: −3.29 to 5.60, *I*^2^ = 0.0%, *P* = 0.831).

### MDP of IDO based on short-term observation at 12 weeks

As shown in Supplementary Table 2, only one RCT [40] was available for MDP. Compared with placebo and results at 6 weeks, 5 different dosages showed no significant declines in MDP. Only 2 of 15 subgroups showed significant differences, i.e., BTX-A 150 U vs. 50 U and 200 U vs. 150 U for IDO (WMD: −8.90, 95% CI: −16.98 to −0.82, *I*^2^ = NA, *P* = NA; WMD: 9.90, 95% CI: 1.03 to 18.77, *I*^2^ = NA, *P* = NA, respectively).

### MDP of IDO based on short-term observation at 36 weeks

Only one RCT [40] reported the effects of different dosages of BTX-A for IDO on MDP at 36 weeks. As shown in Supplementary Table 2, no dose of BTX-A demonstrated effectiveness in treatment of OAB compared with placebo.

### 5. Incontinence quality of life (I-QOL) of NDO at 6 weeks

Three RCTs [36, 37, 41] were available for analysis of I-QOL at 6 weeks. As shown in Supplementary Table 1, both BTX-A 300 U and 200 U for NDO compared with placebo significantly improved the QOL (WMD: 22.10, 95% CI: 17.06 to 27.14, *I*^2^ = NA, *P* = NA; WMD: 16.10, 95% CI: 10.70 to 21.50, *I*^2^ = NA, *P* = NA). However, there were no significant differences in improvement of I-QOL at 6 weeks between BTX-A 300 U and 200 U (WMD: 3.66, 95% CI: −0.35 to 7.67, *I*^2^ = 0.0%, *P* = 0.605).

### Safety

Total adverse events were also reported in 6 studies [33–37, 39] that evaluated the safety of BTX-A injections for OAB. Adverse events included urinary tract infections (UTI), urinary retention, hematuria, and muscle weakness, among others. The subgroup details are shown in Supplementary Tables 1 and 2. The results for 300 U and 200 U vs. placebo for NDO were significant and are clearly apparent in Supplementary Table 1 (BTX-A 300 U vs. placebo, relative risk (RR): 1.13, 95% CI: 1.07 to 1.20, *I*^2^ = 0.0%, *P* = 0.897; BTX-A 200 U vs. placebo, RR: 1.15, 95% CI: 1.09 to 1.21, *I*^2^ = 0.0%, *P* = 0.988). However, significant differences were not observed between BTX-A 300U and 200U (RR: 0.99, 95% CI: 0.94 to 1.03, *I*^2^ = 0.0%, *P* = 0.984). For IDO, as is shown in Supplementary Table 2, there were no significant differences in BTX-A 300U and 200U vs. placebo (BTX-A 300 U vs. placebo, RR: 1.09, 95% CI: 0.89 to 1.33, *I*^2^ = NA, *P* = NA; BTX-A 200 U vs. placebo, RR: 1.10, 95% CI: 0.90 to 1.35, *I*^2^ = NA, *P* = NA) and other subgroups. These data indicated that therapy of NDO with BTX-A 300 U and 200 U leads to more complications than with placebo, while BTX-A for IDO have fewer complications.

## DISCUSSION

OAB is a highly prevalent disease with a detrimental effect on patients, whose quality of life is greatly reduced due to the urinary incontinence caused by OAB [42]. Therefore, the treatment of OAB requires urgent attention. Although various treatment modalities including bladder and behavioral training, biofeedback, electrical stimulation, surgery, and pharmacotherapy [21, 43] have been used for OAB, the results have often been unsatisfactory. For example, anticholinergics are the current first-line pharmacotherapeutic agents indicated for OAB, but no other effective medicines are available; however, these also have unwanted side effects, resulting in poor compliance and a suboptimal response [44, 45]. Hence, a new treatment is needed for OAB. BTX-A has been found to be suitable for both neurogenic and idiopathic incontinence [46, 47], and may also be useful for NDO and IDO.

BTX-A mainly inhibits the release of acetylcholine at nerve terminals and paralyzes the detrusor, thereby improving bladder conditions and reducing urinary symptoms [48]. Based on the theory that nerve terminals develop new acetylcholine-release sites and become functional over 3 to 9 months [49], and the fact that most RCTs repeated injections at more than 12 weeks after the first injection, we defined more than 12 weeks as long-term and not more than 12 weeks as short-term.

In this meta-analysis, the effectiveness and safety of 5 different dosages of BTX-A, including 50 U, 100 U, 150 U, 200 U, and 300 U, as well as placebo, were analyzed. However, placebo and not all dosages were included and compared for analysis of each outcome indicator. This meta-analysis showed that BTX-A 300U and 200U significantly improved symptoms of NDO, while significant differences were not seen in BTX-A 300U and 200U for IDO, compared with placebo. Obvious efficacies were not seen in BTX-A 50U, 100U and 150U for IDO except for BTX-A 100U and 150 U for the volume per void of IDO, compared with placebo. In addition, the efficacies of different dosages of BTX-A were not consistent in short- and long-term observations, and effect differences did not depend on the relationship between different doses for the same outcomes, as for UI episodes per week of NDO at 2 weeks and MCC of IDO at 12 weeks. BTX-A 300 U and 200 U had nearly the obvious effect on all outcomes for NDO in short-term observation (2 and 6 weeks), especially at 6 weeks. In addition, the results for outcomes including UI episodes per week, MCC, and MDP of NDO were consistent with those of other corresponding meta-analyses [15, 21]. Nevertheless, compared with placebo, there were no significant differences in effectiveness for MCC and MDP with BTX-A 300 U and 200 U for IDO at 36 weeks, and the changes in MDP showed no significant differences with BTX-A 300 U and 200 U for IDO at 12 weeks, which may be the result of BTX-A wash-out and targeted areas of BTX-A for NDO and IDO are different or other factors resulting in IDO interfere with the effect of BTX-A. Additionally, BTX-A 300 U and 200 U still remained effective for UI episodes per week at 12 weeks. Compared with placebo, it seemed unusual that BTX-A 200 U showed no significant improvement in MCC of IDO, while BTX-A 150 U and 300 U had opposite effects at 12 weeks; these findings were not consistent with the results of the meta-analysis by Sun et al. [19], in which the same data were used for different dosages in assessing MCC of IDO. This may be the result of small sample sizes and fewer studies included for MCC of IDO in this meta-analysis. Chapple et al [50] reported that treatment with BTX-A 100 U provided both statistically significant and clinically relevant improvements in all OAB symptoms. This meta-analysis also revealed that doses of 100 U and 150U were significantly effective in increasing volume per void of IDO at 12 weeks and doses of 200U and 300U were significantly effective in increasing volume per void of IDO at 12 weeks. To better examine long-term effectiveness and drug tolerance, the outcomes of repeated BTX-A injections were also included for analysis; the results showed that significant improvements in UI episodes per week, MCC, and I-QOL of NDO were present at 6 weeks. Kennelly et al [37] focused on the results of repeated treatment for up to five cycles. Due to all included studies were only survey about UI episodes per week in patients with NDO, weren’t the UI episodes per week in patients with IDO. These meta-analyses can’t survey research the outcome for UI episodes per week in patients with IDO.

Almost all previous systematic reviews and meta-analyses reported adverse reactions such as UTIs, urinary retention, hematuria, and muscle weakness. Therefore, individual adverse reaction was not analyzed in this meta-analysis. However, the total adverse events including urinary retention, hematuria, and muscle weakness, among others were not reported in previous meta-analysis [15–17, 19–21]. Hence, the total adverse events were analyzed to evaluate the safety of BTX-A for NDO and OAB. We found that BTX-A 300 U and 200 U for NDO had slightly more adverse events than placebo, and for IDO had fewer adverse events. In addition, dosages less than 200U of BTX-A for IDO also had fewer adverse events. However, treatment with BTX-A was well-tolerated and adverse effects were mainly limited to localized urologic events including UTIs, urinary retention and hematuria, which were easily treated and managed. Other adverse effects were also well-tolerated.

The highlights of this meta-analysis are as follows. First, different doses of BTX-A were evaluated, and the effectiveness of BTX-A was assessed for multiple outcomes including symptoms, bladder function, and incontinence quality of life (I-QOL). Second, we comprehensively and systematically evaluated the effectiveness of BTX-A for two types of OAB including NDO and IDO from two observation periods, and also focused on the safety of BTX-A, thereby reducing the risk of errors from correct outcomes. Furthermore, subgroup analyses of dosages and types of OAB were performed to substitute for pooled dosages or comparisons of grouped dosages from different studies, to objectively reflect true effectiveness and safety, and to guide clinicians to treat two kinds of OAB. However, there are also several limitations which should be discussed in our meta-analysis. First, only 11 RCTs with insufficient numbers of patients were included in our meta-analysis, which may influence our conclusions. Second, benign prostatic hyperplasia (BPH) is a prominent disease in males. Although the pathogenesis of OAB in males and females is different, subgroup analyses for pathogenesis were not performed in this meta-analysis. With few RCTs and lack of relevant data, more studies are needed to investigate male and female differences. Third, although this meta-analysis assessed the effectiveness of BTX-A from two observation periods, the short- and long-term observations were insufficient. Therefore, short- and long-term observations and the use of repeated injections require further investigations. In addition, more high-quality RCTs with larger sample sizes should be performed, and a subsequent updated meta-analysis will be necessary for in-depth and comprehensive assessment of the safety and efficiency of BTX-A.

## MATERIALS AND METHODS

This meta-analysis was conducted according to the Preferred Reporting Items for Systematic Reviews and Meta-analyses (PRISMA) statement [51]. No ethical issues were involved in this study, and all collected data were based on published studies.

### Search strategy

The MEDLINE, EMBASE, and Cochrane Controlled Trials Register databases were searched through November 3, 2016 to identify relevant articles that evaluated the effectiveness and safety of BTX-A for treatment of OAB. We also searched the reference lists of the retrieved studies. The search keywords were as follows: “urinary bladder”, “overactive bladder”, “overactive detrusor”, “overactive detrusor function”, “detrusor overactive”, “urinary incontinence”, “botulinum toxin”, “Clostridium botulinum”, “onabotulinumtoxin”, “abobotulinumtoxin”, and “BOTOX”.

### Inclusion and exclusion criteria and trial selection

RCTs were included for meta-analysis if the following criteria were met: (1) all patients were diagnosed with urinary incontinence due to OAB, (2) the study population was over 18 years old, (3) the studies compared BTX-A with placebo or different dosages of BTX-A, and (4) the language was limited to English.

Articles were excluded for the following reasons: (1) patients had stress incontinence, (2) the study was a duplicate, (3) the data could not be extracted or obtained through contact with the author, or (4) insufficient information was available to calculate missing standard deviations (SD) for continuous outcomes.

### Quality assessment

The quality of the studies was assessed according to random sequence generation, allocation concealment, blinding of participants and personnel, blinding of outcome assessment, incomplete outcome data, and selective reporting and other biases. In accordance with the Cochrane Handbook for Systematic Reviews of Interventions v.5.1.0 [52], and based on the quality assessment criteria, each study was rated and assigned to one of the three following categories: if all quality criteria were adequately met, the study was deemed to have a low risk of bias; if one or more quality criteria were unclear, the study was deemed to have an unclear risk of bias; if one or more of the criteria was not met or not included, the study was deemed to have a high risk of bias. Differences were discussed among the authors.

### Data collection

We carefully reviewed the titles, abstracts, and the full texts of the articles. The following information in each study was collected for our meta-analysis: (1) basic information of the study and patients: the author, year, sample size, and design of each study; the gender, ages, type of urinary incontinence, and basic disease; (2) method: the details of the treatment group and the control group of each study; (3) outcome: UI episodes per week as the primary outcome, and urodynamic parameters including MCC, MDP, and the volume per void as the secondary outcomes; the I-QOL of patients and the total adverse events. The time points of study outcomes were approximately divided into two periods, based on Frenkl’s study [49]. We defined time points as follows: not more than 12 weeks, short-term observation; more than 12 weeks, long-term observation. Data for single cycle injections and repeated injections were collected for this meta-analysis.

All data were independently collected by two reviewers. Disagreements on the extracted data were resolved by discussion and consultation with an expert.

### Statistical analysis

This meta-analysis was performed using Stata 12.0. Dichotomous and continuous outcomes were expressed as relative risk (RR) with 95% confidence interval (CI) [52, 53], and weighted mean difference (WMD) [52] with 95% CI, at a significance level of *P* < 0.05, respectively. Heterogeneity [54] between studies was also evaluated and measured using chi-square tests, at a significance level of *P* < 0.1. *I*^2^ = 0 indicates no heterogeneity and larger values of *I*^2^ represent greater heterogeneity. When *I*^2^ > 50%, studies were considered to have significant heterogeneity; we then used a random-effects model to conduct the meta-analysis. For *I*^2^ < 50%, a fixed-effects model was used [52]. Because dosages and the types of OAB were causes of heterogeneity, subgroup analysis for dosages and types of OAB including IDO and NDO was performed.

## CONCLUSIONS

This meta-analysis suggests that BTX-A 300 U and 200U have significant efficacies for NDO for short-term treatments, while obvious efficacies were not seen in BTX-A 300 U and 200 U for IDO for long-term treatments. Obvious efficacies were also not seen in BTX-A 50 U, 100U and 150U for IDO except for BTX-A 100 U and 150 U for the volume per void of IDO at 12 weeks. In addition, the treatments of BTX-A were with minimal, local, and manageable adverse effects. Hence, BTX-A 200 U is recommended for management of NDO for short-term treatment for there was no significant difference from the larger dose of 300U. The short-term efficacies of BTX-A for IDO remain to be investigated.

### Ethical approval

Not needed.

## SUPPLEMENTARY MATERIALS TABLES




