# Supplementary material for: A systematic review and meta-analysis of effectiveness and safety of therapy for overactive bladder using botulinum toxin A at different dosages
Source: Oncotarget. 2017 Aug 7;8(52):90338–50. doi: 10.18632/oncotarget.20056 (PMC5685754; doi:10.18632/oncotarget.20056)
Supplement: Supplementary file 1 [file oncotarget-08-90338-s001.pdf]

A systematic review and meta-analysis of effectiveness and safety of therapy for overactive bladder using botulinum toxin a at different dosages

SUPPLEMENTARY MATERIALS

Supplementary Table 1: Summary of result with different dosage at different observation point for NDO

Outcomes	Number of RCTs	WMD/RR	95% CI	I^2 (%)	P_{het}
**Urinary Incontinence (UI) Episodes per Week**					
**2 weeks**					
BTX-A 200U VS. Placebo	1	-9.10	-14.10, -4.10	NA	NA
BTX-A 300U VS. Placebo	1	-6.10	-12.54, 0.34	NA	NA
BTX-A 300U VS. 200U	1	3.00	-3.30, 9.30	NA	NA
**6 weeks**					
BTX-A 200U VS. Placebo	3	-10.72	-13.40, -8.04	0.0	0.626
BTX-A 300U VS. Placebo	3	-11.42	-13.91, -8.93	50	0.135
BTX-A 300U VS. 200U	4	-0.38	-2.60, 1.84	0.0	0.765
**12 weeks**					
BTX-A 200U VS. Placebo	1	-8.50	-14.46, -2.54	NA	NA
BTX-A 300U VS. Placebo	1	-7.80	-13.73, -1.87	NA	NA
BTX-A 300U VS. 200U	1	0.70	-4.73, 6.13	NA	NA
**Maximum Cystometric Capacity (MCC)**					
**6 weeks**					
BTX-A 200U VS. Placebo	5	141.45	123.57, 159.33	0.0	0.991
BTX-A 300U VS. Placebo	5	151.52	132.86, 170.18	0.0	0.990
BTX-A 300U VS. 200U	6	9.23	-11.61, 30.06	0.0	0.988
**Volume per Void**					
**2 weeks**					
BTX-A 200U VS. Placebo	1	42.60	14.49, 70.71	NA	NA
BTX-A 300U VS. Placebo	1	49.90	17.54, 82.26	NA	NA
BTX-A 300U VS. 200U	1	7.30	-30.28, 44.88	NA	NA
**6 weeks**					
BTX-A 200U VS. Placebo	2	92.68	76.22, 109.14	0.0	0.982
BTX-A 300U VS. Placebo	2	100.73	83.24,118.22	0.0	0.328
BTX-A 300U VS. 200U	3	4.72	-12.12, 21.57	0.0	0.663
**12 weeks**					
BTX-A 200U VS. Placebo	1	83.70	54.54, 112.86	NA	NA
BTX-A 300U VS. Placebo	1	78.80	46.23, 111.37	NA	NA
BTX-A 300U VS. 200U	1	-4.90	-43.16, 33.36	NA	NA
**Maximum Detrusor Pressure (MDP)**					
**6 weeks**					
BTX-A 200U VS. Placebo	5	-33.01	-37.75, -28.27	0.0	0.998
BTX-A 300U VS. Placebo	5	-31.31	-35.79, -26.84	0.0	0.679
BTX-A 300U VS. 200U	6	1.16	-3.29, 5.60	0.0	0.831
**Incontinence Quality of Life (I-QoL)**					
**6 weeks**					
BTX-A 200U VS. Placebo	1	16.10	10.70, 21.50	NA	NA
BTX-A 300U VS. Placebo	1	22.10	17.06, 27.14	NA	NA
BTX-A 300U VS. 200U	3	3.66	-0.35, 7.67	0.0	0.605
**Total Adverse Events***					
BTX-A 200U VS. Placebo	5	1.15	1.09, 1.21	0.0	0.988
BTX-A 300U VS. Placebo	5	1.13	1.07, 1.20	0.0	0.897
BTX-A 300U VS. 200U	6	0.99	0.94,1.03	0.0	0.984

P_{het}: Test of heterogeneity, *: The effect size of RR was only employed at outcome of total adverse events, NA: Not available.

Supplementary Table 2: Summary of result with different dosage at different observation point for IOAB. See Supplementary_Table_2
