# Supplementary material for: A systematic review and meta-analysis of effectiveness and safety of therapy for overactive bladder using botulinum toxin A at different dosages
Source: Oncotarget. 2017 Aug 7;8(52):90338–50. doi: 10.18632/oncotarget.20056 (PMC5685754; doi:10.18632/oncotarget.20056)
Supplement: Supplementary file 2 [file oncotarget-08-90338-s002.docx]

**Supplementary Table 2: Summary of result with different dosage at different observation point for IOAB**

| Outcomes | Number of RCTs | WMD/  RR | 95% CI | I^2^ (%) | P_het_ |
| --- | --- | --- | --- | --- | --- |
| **Maximum cystometric capacity (MCC)** | | | | | |
| **12 weeks** |  |  |  |  |  |
| BTX-A 50U VS. Placebo | 2 | 7.13 | -32.75, 47.00 | 0.0 | 0.714 |
| BTX-A 100U VS. Placebo | 2 | 36.95 | -6.73, 80.64 | 0.0 | 0.372 |
| BTX-A 150U VS. Placebo | 2 | 58.92 | 16.02, 101.81 | 0.0 | 0.715 |
| BTX-A 200U VS. Placebo | 1 | 42.00 | -13.50, 97.50 | NA | NA |
| BTX-A 300U VS. Placebo | 1 | 81.30 | 26.21, 136.39 | NA | NA |
| BTX-A 100U VS. 50U | 2 | 28.29 | -11.03, 67.60 | 0.0 | 0.559 |
| BTX-A 150U VS. 50U | 2 | 52.08 | 12.93, 91.22 | 0.0 | 0.978 |
| BTX-A 150U VS. 100U | 2 | 23.81 | -18.19, 65.81 | 0.0 | 0.603 |
| BTX-A 200U VS. 50U | 1 | 41.50 | -5.04, 88.04 | NA | NA |
| BTX-A 200U VS. 100U | 2 | 13.02 | -29.64, 55.68 | 0.0 | 0.534 |
| BTX-A 200U VS. 150U | 1 | -10.20 | -59.81, 39.41 | NA | NA |
| BTX-A 300U VS. 50U | 1 | 80.80 | 34.74, 126.86 | NA | NA |
| BTX-A 300U VS. 100U | 1 | 59.80 | 11.53,108.07 | NA | NA |
| BTX-A 300U VS. 150U | 1 | 29.10 | -20.06, 78.26 | NA | NA |
| BTX-A 300U VS. 200U | 1 | 39.30 | -9.20, 87.80 | NA | NA |
| **36 weeks** |  |  |  |  |  |
| BTX-A 50U VS. Placebo | 1 | 8.50 | -61.02, 78.02 | NA | NA |
| BTX-A 100U VS. Placebo | 1 | 25.90 | -44.52, 96.32 | NA | NA |
| BTX-A 150U VS. Placebo | 1 | 37.30 | -32.74, 107.34 | NA | NA |
| BTX-A 200U VS. Placebo | 1 | 35.70 | -36.55, 107.95 | NA | NA |
| BTX-A 300U VS. Placebo | 1 | 46.80 | -22.97, 116.57 | NA | NA |
| BTX-A 100U VS. 50U | 1 | 17.40 | -34.93, 69.73 | NA | NA |
| BTX-A 150U VS. 50U | 1 | 28.80 | -23.01, 80.61 | NA | NA |
| BTX-A 150U VS. 100U | 1 | 11.40 | -41.62, 64.42 | NA | NA |
| BTX-A 200U VS. 50U | 1 | 27.20 | -27.56, 81.96 | NA | NA |
| BTX-A 200U VS. 100U | 1 | 35.59 | -6.41, 77.58 | NA | NA |
| BTX-A 200U VS. 150U | 1 | -1.60 | -57.01,53.81 | NA | NA |
| BTX-A 300U VS. 50U | 1 | 38.30 | -13.14, 89.74 | NA | NA |
| BTX-A 300U VS. 100U | 1 | 20.90 | -31.76, 73.56 | NA | NA |
| BTX-A 300U VS. 150U | 1 | 9.50 | -42.65, 61.65 | NA | NA |
| BTX-A 300U VS. 200U | 1 | 11.10 | -43.97, 66.17 | NA | NA |
| **Volume per Void** | | | | | |
| **12 Weeks** |  |  |  |  |  |
| BTX-A 50U VS. Placebo | 1 | 46.00 | -24.60, 116.60 | NA | NA |
| BTX-A 100U VS. Placebo | 2 | 34.48 | 16.73, 52.23 | 42.0 | 0.189 |
| BTX-A 150U VS. Placebo | 1 | 81.00 | 7.98, 154.02 | NA | NA |
| BTX-A 100U VS. 50U | 1 | 34.40 | 3.79, 65.01 | NA | NA |
| BTX-A 150U VS. 50U | 1 | 35.00 | -0.43,70.43 | NA | NA |
| BTX-A 150U VS. 100U | 1 | 0.60 | -35.25, 36.45 | NA | NA |
| **Maximum Detrusor Pressure (MDP)** | | | | | |
| **12 weeks** |  |  |  |  |  |
| BTX-A 50U VS. Placebo | 1 | 4.70 | -3.49, 12.89 | NA | NA |
| BTX-A 100U VS. Placebo | 1 | 0.20 | -7.55, 7.95 | NA | NA |
| BTX-A 150U VS. Placebo | 1 | -4.20 | -12.70, 4.30 | NA | NA |
| BTX-A 200U VS. Placebo | 1 | 5.70 | -3.28, 14.68 | NA | NA |
| BTX-A 300U VS. Placebo | 1 | 0.10 | -9.86, 10.06 | NA | NA |
| BTX-A 100U VS. 50U | 1 | -4.50 | -11.78,2.78 | NA | NA |
| BTX-A 150U VS. 50U | 1 | -8.90 | -16.98, -0.82 | NA | NA |
| BTX-A 150U VS. 100U | 1 | -4.40 | -12.03, 3.23 | NA | NA |
| BTX-A 200U VS. 50U | 1 | 1.00 | -7.57,9.57 | NA | NA |
| BTX-A 200U VS. 100U | 1 | 5.50 | -2.66, 13.66 | NA | NA |
| BTX-A 200U VS. 150U | 1 | 9.90 | 1.03, 18.77 | NA | NA |
| BTX-A 300U VS. 50U | 1 | -4.60 | -14.20, 5.00 | NA | NA |
| BTX-A 300U VS. 100U | 1 | -0.10 | -9.33, 9.13 | NA | NA |
| BTX-A 300U VS. 150U | 1 | 4.30 | -5.57, 14.17 | NA | NA |
| BTX-A 300U VS. 200U | 1 | -5.60 | -15.88, 4.68 | NA | NA |
| **36 weeks** |  |  |  |  |  |
| BTX-A 50U VS. Placebo | 1 | 2.60 | -8.24, 13.44 | NA | NA |
| BTX-A 100U VS. Placebo | 1 | 8.00 | -2.52, 18.52 | NA | NA |
| BTX-A 150U VS. Placebo | 1 | 3.40 | -7.14, 13.94 | NA | NA |
| BTX-A 200U VS. Placebo | 1 | 3.80 | -6.66, 14.26 | NA | NA |
| BTX-A 300U VS. Placebo | 1 | 8.10 | -3.15, 19.35 | NA | NA |
| BTX-A 100U VS. 50U | 1 | 5.40 | -1.62,12.42 | NA | NA |
| BTX-A 150U VS. 50U | 1 | 0.80 | -6.25, 7.85 | NA | NA |
| BTX-A 150U VS. 100U | 1 | -4.60 | -11.15, 1.95 | NA | NA |
| BTX-A 200U VS. 50U | 1 | 1.20 | -5.73,8.13 | NA | NA |
| BTX-A 200U VS. 100U | 1 | -4.20 | -10.62, 2.22 | NA | NA |
| BTX-A 200U VS. 150U | 1 | 0.40 | -6.05, 6.85 | NA | NA |
| BTX-A 300U VS. 50U | 1 | 5.50 | -2.58,13.58 | NA | NA |
| BTX-A 300U VS. 100U | 1 | 0.10 | -7.55, 7.75 | NA | NA |
| BTX-A 300U VS. 150U | 1 | 4.70 | -2.97, 12.37 | NA | NA |
| BTX-A 300U VS. 200U | 1 | 4.30 | -3.26, 11.86 | NA | NA |
| **Total Adverse Events*** | | | | | |
| BTX-A 50U VS. Placebo | 1 | 1.02 | 0.83, 1.27 | NA | NA |
| BTX-A 100U VS. Placebo | 1 | 1.04 | 0.84, 1.29 | NA | NA |
| BTX-A 150U VS. Placebo | 1 | 1.02 | 0.82, 1.27 | NA | NA |
| BTX-A 200U VS. Placebo | 1 | 1.10 | 0.90,1.35 | NA | NA |
| BTX-A 300U VS. Placebo | 1 | 1.09 | 0.89,1.33 | NA | NA |
| BTX-A 100U VS. 50U | 1 | 1.02 | 0.84,1.23 | NA | NA |
| BTX-A 150U VS. 50U | 1 | 0.99 | 0.81,1.21 | NA | NA |
| BTX-A 150U VS. 100U | 1 | 0.98 | 0.80, 1.19 | NA | NA |
| BTX-A 200U VS. 50U | 1 | 1.08 | 0.90, 1.29 | NA | NA |
| BTX-A 200U VS. 100U | 1 | 1.06 | 0.89, 1.26 | NA | NA |
| BTX-A 200U VS. 150U | 1 | 1.08 | 0.90, 1.31 | NA | NA |
| BTX-A 300U VS. 50U | 1 | 1.06 | 0.89, 1.27 | NA | NA |
| BTX-A 300U VS. 100U | 1 | 1.05 | 0.88, 1.25 | NA | NA |
| BTX-A 300U VS. 150U | 1 | 10.7 | 0.89, 1.29 | NA | NA |
| BTX-A 300U VS. 200U | 1 | 0.99 | 0.84, 1.17 | NA | NA |

P_het_: Test of heterogeneity, *: The effect size of RR was only employed at outcome of total adverse events, NA: Not available.
